# Rapid microsatellite development for tree peony and its implications

**DOI:** 10.1186/1471-2164-14-886

**Published:** 2013-12-16

**Authors:** Zhimin Gao, Jie Wu, Zheng’an Liu, Liangsheng Wang, Hongxu Ren, Qingyan Shu

**Affiliations:** 1 Beijing Botanical Garden, Key Laboratory of Plant Resources, Institute of Botany, The Chinese Academy of Sciences, Beijing 100093, PR China; 2 International Center for Bamboo and Rattan, Key Laboratory on the Science and Technology of Bamboo and Rattan, Beijing 100102, PR China; 3 University of Chinese Academy of Sciences, Beijing 100049, PR China

**Keywords:** zzzMicrosatellite, Next-generation sequencing, Tree peozzzzny, Ornamental, SSR marker

## Abstract

**Background:**

Microsatellites are ubiquitous in genomes of various organisms. With the realization that they play roles in developmental and physiological processes, rather than exist as ‘junk’ DNA, microsatellites are receiving increasing attention. Next-generation sequencing allows acquisition of large-scale microsatellite information, and is especially useful for plants without reference genome sequences.

**Results:**

In this study, enriched DNA libraries of tree peony, a well-known ornamental woody shrub, were used for high-throughput microsatellite development by 454 GS-FLX Titanium pyrosequencing. We obtained 675,221 reads with an average length of 356 bp. The total size of examined sequences was 240,672,018 bp, from which 237,134 SSRs were identified. Of these sequences, 164,043 contained SSRs, with 27% featuring more than one SSR. Interestingly, a high proportion of SSRs (43%) were present in compound formation. SSRs with repeat motifs of 1–4 bp (mono-, di-, tri-, and tetra-nucleotide repeats) accounted for 99.8% of SSRs. Di-nucleotide repeats were the most abundant. As in most plants, the predominant motif in tree peony was (A/T)_n_, with (G/C)_n_ less common. The lengths of SSRs were classified into 11 groups. The shortest SSRs (10 bp) represented 1% of the total number, whereas SSRs 21–30 and 101–110 bp long accounted for 26% and 29%, respectively, of all SSRs. Many sequences (42,111) were mapped to CDS (coding domain sequence) regions using *Arabidopsis* as a reference. GO annotation analysis predicted that CDSs with SSRs performed various functions associated with cellular components, molecular functions, and biological processes. Of 100 validated primer pairs, 24 were selected for polymorphism analysis among 23 genotypes; cluster analysis of the resulting data grouped genotypes according to known relationships, confirming the usefulness of the developed SSR markers.

**Conclusions:**

The results of our large-scale SSR marker development using tree peony are valuable for investigating plant genomic structural evolution and elucidating phenotypic variation in this species during its evolution and artificial selection. The newly identified SSRs should be useful for genetic linkage map construction, QTL mapping, gene location and cloning, and molecular marker-assisted breeding. In addition, the genome-wide marker resources generated in this study should aid genomic studies of tree peony and related species.

## Background

Microsatellites, or simple sequence repeats (SSRs), are tandemly repeated 1–6-bp DNA regions ubiquitous in prokaryotes and eukaryotes. As components of genomes, they can be found both in protein-coding and non-coding regions. SSRs have been universally utilized as genetic markers because of their abundance and inherent potential for variation [1]. The functions of SSRs were previously unclear, and until recently they were regarded as ‘junk’ (i.e., having no significant genomic role). At present, much progress has been achieved in regard to elucidation of SSR function. SSR locations appear to determine the types of functional roles that SSRs play, and alterations in SSR lengths at different locations can lead to changes in organismal phenotypes [2,3]. SSRs in different gene positions (i.e., promoter regions, 5′ untranslated regions (UTRs), 3′ UTRs, exons, and introns) may play important roles in determining protein function, genetic development, and regulation of gene expression. For example, expansion of CAG repeats in the *HD* gene coding region can lead to Huntington’s disease in humans, possibly through activation of some so-called ‘toxic’ proteins [1]. With expanding knowledge of SSR functions in terms of development, gene regulation, and evolution, SSRs are receiving increasing attention. Because genomic information is lacking for most species, however, it is difficult to study microsatellite origin, distribution, and evolution, or even to develop new SSR-based molecular markers.

Traditional SSR development is time-consuming, and involves laborious iterations of genomic DNA library screening with SSR probes required to isolate microsatellite-containing sequences [4]. Next-generation sequencing technologies are remarkably well-developed, and are widely used for genome sequencing, transcriptome sequencing, and genome deep-sequencing in plants [5,6]. It has been successfully used for identifying molecular markers, including SSRs and simple nucleotide polymorphisms (SNPs), in organisms such as the water strider [7], copperhead snake [8], blue duck [9], pine pathogen fungus [10], and scuttle fly [11]. Because of the complicated structure of plant genomes, however, molecular marker development using next-generation sequencing has had limited application, especially in non-model plants lacking genomic information.

Among next generation-sequencing approaches, Roche 454 pyrosequencing (R454) holds great promise with respect to the long reads obtained as well as acquisition of sufficient genetic information of interest within single reads. The large amount of generated data facilitates sequence assembly without genomic information [5], and increases the likelihood that a single read contains microsatellite repeats along with suitable flanking regions of unique sequences. Another approach to mining molecular markers, involving *in silico* methods, has also been successful; examples include the derivation of markers from a draft genome [12] and the mining of existing expressed sequence tag (EST) libraries [13]. Compared with traditional library-based and *in silico* methods, R454 offers great advantages, being faster, less costly, and less dependent on existing genetic resources [14]. Another advantage is the huge amount of genetic information produced, with the possibility of future use. This is greatly beneficial for studies of plants without genomic information, especially woody plants; in such species, no established systems exist for *in vitro* culture or transformation for genetic manipulation, hampering new cultivar breeding. Molecular marker-assisted breeding is efficient for such organisms. DNA markers developed via next-generation sequencing are also increasingly being used for genetic diagnostics, drug discovery, gene cloning, genome analysis, comparative genomics, and molecular evolution studies.

The purpose of this study was to apply next-generation sequencing, such as R454, to SSR development in tree peony (*Paeonia suffruticosa* Andrews). Such an approach was expected to drastically shorten the time required for effective marker development and utilization. Tree peony belongs to sect. *Moutan* DC. of the genus *Paeonia* L. (*Paeoniaceae*). It is a well-known ornamental plant enjoying worldwide popularity on account of its large, showy, colorful and fragrant flowers. Little genomic information is currently available for this species. In a previous study, we constructed a cDNA library from flower buds and obtained 2,241 ESTs, from which 167 SSRs were derived and a dataset of 185 putative SNPs obtained for breeding based on their high availability and stability [15]. Although more than 200 SSRs have been submitted to public databases [16], the number is inadequate with respect to the 1,500 cultivars of tree peony. Compared with crop plants such as maize, wheat, and soybean, or ornamental plants such as rose, molecular markers, especially SSRs, are still needed for future breeding of tree peony. This is especially true taking into consideration its importance, barely transformed nature, and woody characteristics, and the lack of genomic information. Construction of a dense genetic linkage map and development of genome-wide molecular markers are also essential for marker-assisted selection of new tree peony hybrids. Because relationships among wild species of tree peony and their taxonomic position within *Paeoniaceae* are still unclear, developed polymorphic SSRs would also aid studies of *Paeonia* evolution, comparative genetics, and population structure.

## Results

### Sequencing and characterization of reads

**Figure 1:**
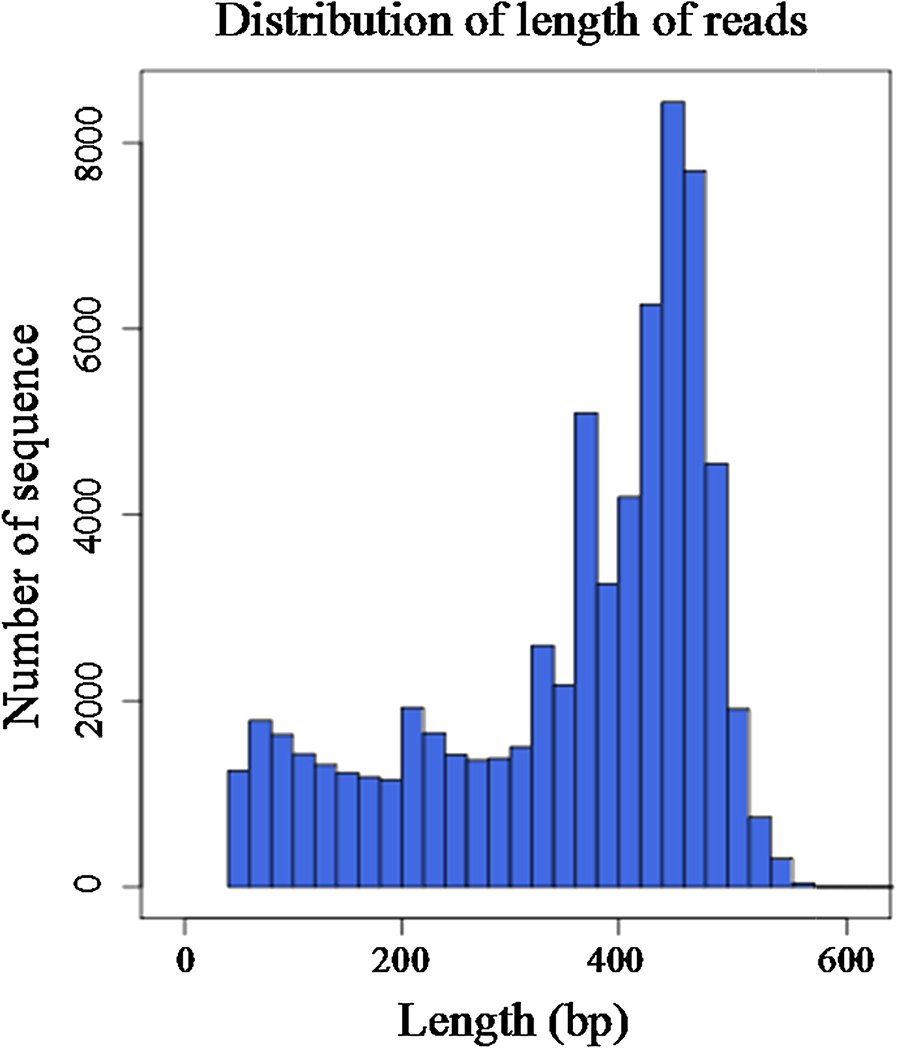
**Length distribution of 454 sequencing reads.** X- and y-axes refer respectively to sequence length in bp and the number of sequences of a given length.

R454 sequencing of the tree peony library generated 675,221 reads averaging 356 bp long, with a maximum length of 590 bp (Figure 1). Among these nucleotides, adenine was the most abundant (30.4%), followed by cytosine (26.7%), thymine (23.5%), and guanine (19.4%). G + C content was 46.1%. Clean reads were deposited in the NCBI public database (Accession number: SRA098186).

### Identification of SSR loci

**Figure 2:**
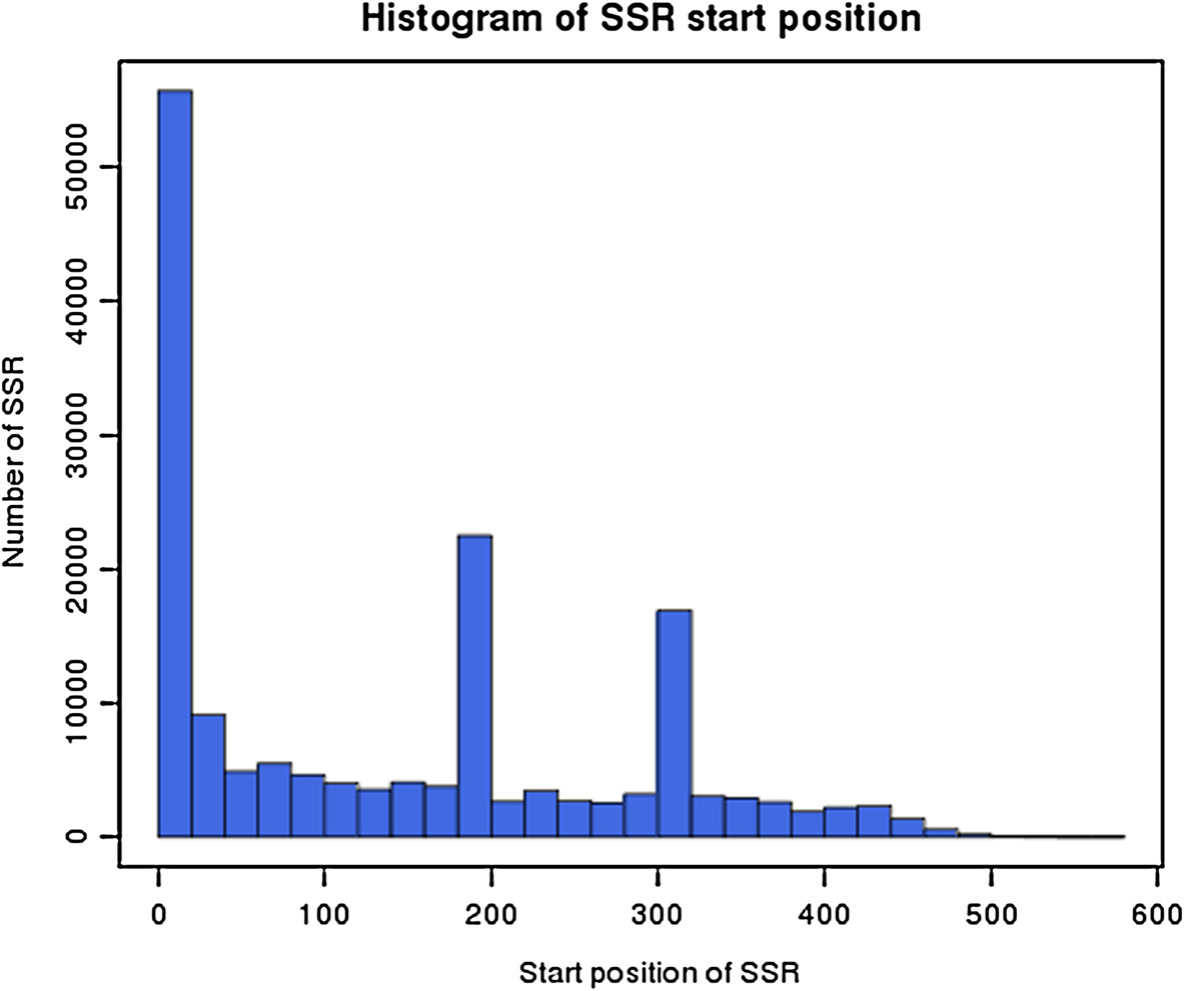
**Distribution of SSR start positions from the 5′-terminus of the cloned library insert.** The x-axis indicates the number of bp from the 5′ terminus of a sequence to the SSR start site. The y-axis corresponds to the number of SSRs beginning at that start position.

**Figure 3:**
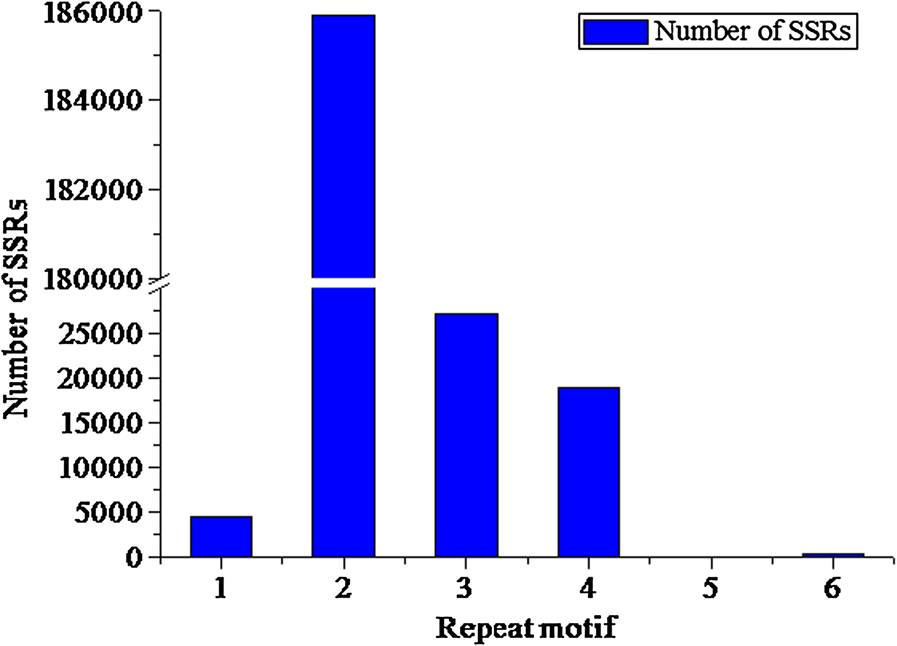
**Total numbers of each repeat motif.** The x-axis indicates different repeat motif. The y-axis indicates the number of SSRs with various repeat motifs. 1: mono- nucleotide repeats; 2: di- nucleotide repeats; 3: tri- nucleotide repeats; 4: tetra- nucleotide repeats; 5: penta- nucleotide repeats; 6: hexa- nucleotide repeats.

MISA was used to analyze a total of 240,672,018 bp of sequences, from which 237,134 SSRs were identified. Of the examined sequences, 164,043 contained SSRs; 27% harbored more than one SSR, with a high proportion of SSRs (43%) present in compound formation (Table 1). The distribution of identified SSR motifs in the cloned sequences was nearly evenly divided between the 400-bp 5′-terminus region and the remaining region outside the 5′-terminus (Figure 2). SSRs with repeat motifs of 1 to 4 bp (mono-, di-, tri-, and tetra-nucleotides) accounted for 99.8% of the total, with di-nucleotide repeats the most abundant (Table 2; Figure 3). SSRs with mono-nucleotide repeats accounted for only 2% of SSRs in tree peony DNA. Proportions of tri-nucleotide and tetra-nucleotide repeats were almost equal, with the combined number of tetra-, penta-, and hexa-nucleotide repeats accounting for at most 8.2% of SSRs.

**Table 1:** Occurrence of microsatellites in the surveyed tree peony genome

**Category**	**Number**
Total number of sequences examined	675221
Total size of examined sequences (bp)	240672018
Total number of identified SSRs	237134
Number of SSR-containing sequences	164043
Number of sequences containing more than 1 SSR	44362
Number of SSRs present in compound formation	70570

**Table 2:** Microsatellite motif length distribution

**Plant species**	**Number of motif repeats**						**Total**	**Percent**
	**Mono**	**Di-**	**Tri-**	**Tetra-**	**Penta-**	**Hexa-**		
*B. distachyon*	30,573	9,407	10625	990	196	84	51875	2.45
*S. bicolor*	55,906	38,138	28480	5368	946	726	129564	5.4
*O. sativa*	64,734	37,282	29189	2565	604	261	135265	2.54
*A. thaliana*	34,843	9386	5596	169	41	57	50092	0.53
*M. truncatula*	1,20,383	20999	9647	1079	216	137	152461	0.94
*P. trichocarpa*	194,557	54304	25130	3178	772	665	278606	1.66
***P. suffruticosa***	**4560**	**185911**	**27235**	**18953**	**99**	**376**	**237134**	**--**

Data for *B. distachyon, S. bicolor, O. sativa, A. thaliana, M. truncatula*, and *P. trichocarpa* were obtained from Sonah et al. 2011 (PLoS One, 6: 1–9).

--: the percent was not calculated due to the Genome of *P. suffruticosa* was unkown.

### Relative frequency of different SSR repeat motifs

A summary of SSRs, including repeat motif and total number of different repeat motifs, is shown in Table 3. Of the two possible types of mono-nucleotide repeats, the most abundant was (A/T)_n_, as in most plants; (G/C)_n_ was much less common in tree peony, accounting for only 0.05% of total SSRs. SSR frequency decreased with increasing motif length (mono- to hexa-nucleotide repeats); most SSRs were composed of mono-, di-, tri-, or tetra- nucleotide repeats, with only a very small share contributed by penta- and hexa-nucleotide repeats. The di-nucleotide repeat (AC)_n_ was more common than (AG)_n_ and (AT)_n_. With respect to tri-nucleotide repeats, A/T-rich repeats were dominant in tree peony (Table 3), with AAC/GTT the most abundant tri-nucleotide motif (65.7%) followed by AAG/CTT (20%). The repeats CCG and ACG were less frequent or absent. The most frequent penta- and hexa-nucleotide repeat motifs were sequences containing the di-nucleotide CpG: AACGT/ACGTT and AAGGAG/CCTTCT, respectively.

**Table 3:** Frequency of mono-, di-, tri-, and tetra-nucleotide repeat motifs in the tree peony genome

**Repeat type**	**Tree peony genome**
**Mono-nucleotide**	**4560**
A/T	3956
C/G	604
**Di-nucleotide**	**185911**
AC/GT	124208
AG/CT	59711
AT/AT	1868
CG/CG	124
**Tri-nucleotide**	**27235**
AAC/GTT	17890
AAG/CTT	5394
AAT/ATT	106
ACC/GGT	1756
ACG/CGT	369
ACT/ATG	606
AGC/CGT	579
AGG/CCT	268
ATC/AGT	262
CCG/CGG	5

### Relative frequencies of different SSR repeat lengths

**Figure 4:**
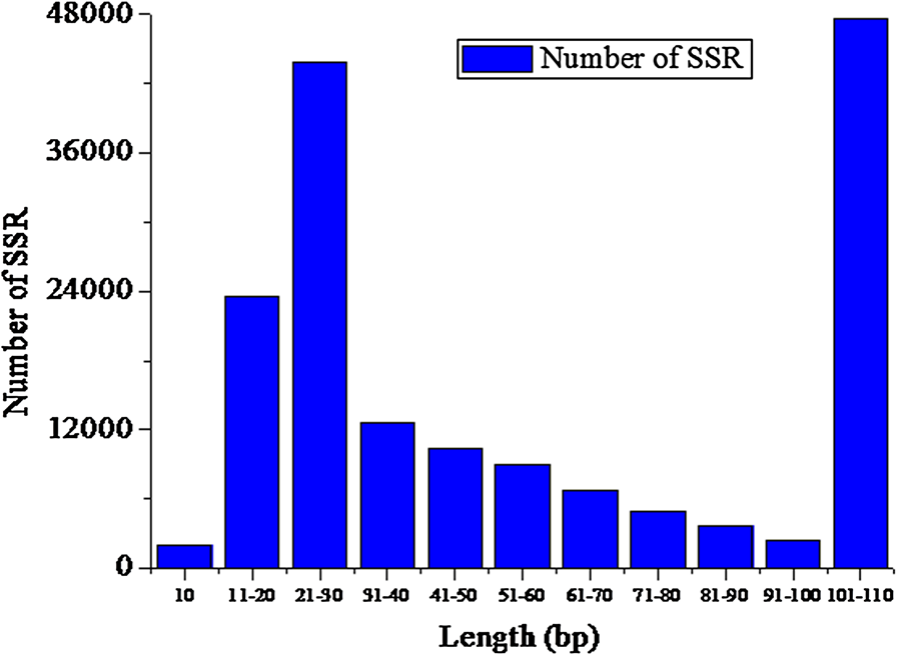
**SSR length distribution.** The x-axis indicates the length of SSRs (bp). The y-axis indicates the number of SSRs with different length.

The lengths of SSRs were classified into 11 groups (Figure 4). The shortest SSRs (10 bp) constituted 1% of the total. SSRs with lengths of 21–30 and 101–110 bp accounted for 26% and 29% of SSRs, respectively (Figure 4). Among di-nucleotide SSRs, the most abundant repeated length was 28 bp, followed by 12 bp and then 30 or 14 bp. The most common length of tri-nucleotide SSRs was approximately 15 bp, with smaller numbers of 18- and 21-bp sequences. Repeat lengths of tetra-nucleotide SSRs ranged from about 20–28 bp.

### Compound SSR analysis

**Figure 5:**
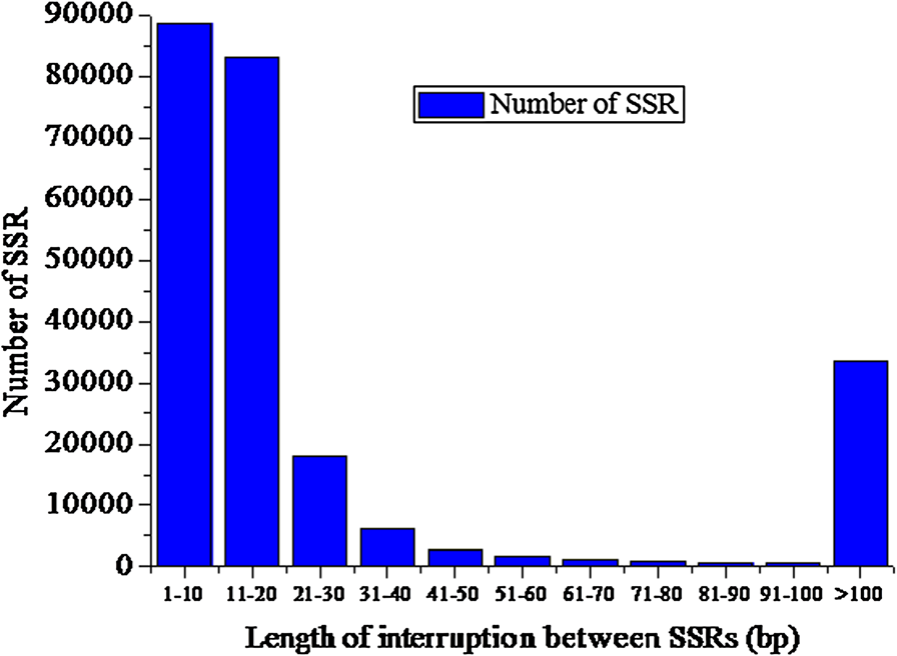
**Distribution of compound SSR interruption distances.** The X-axis indicates the length of interruption between SSRs (bp). The y-axis indicates the number of SSRs with different interruption length.

About 26% of identified SSRs were compound. Interruption distance ranged from 5–195 bp, with most interruptions 5–20 bp long (Figure 5). Many of the compound SSRs were composite, being made up of various combinations of mono- to hexa-nucleotide repeats, such as (mono-nucleotide repeat)_n_-(tetra-nucleotide repeat)_n_, (tetra-nucleotide repeat)_n_-(tetra-nucleotide repeat)_n_, (mono-nucleotide repeat)_n_-(tri-nucleotide repeat)_n_, (tri-nucleotide repeat)_n_-(tri-nucleotide repeat)_n_, or (hexa-nucleotide repeat)_n_-(tri-nucleotide repeat)_n_. About 56% of repeat motifs were found in compound SSRs, revealing the complexity of the tree peony genome.

### Microsatellite distribution in different genomic regions of tree peony using *Arabidopsis*, poplar, and grape reference sequences

**Figure 6:**
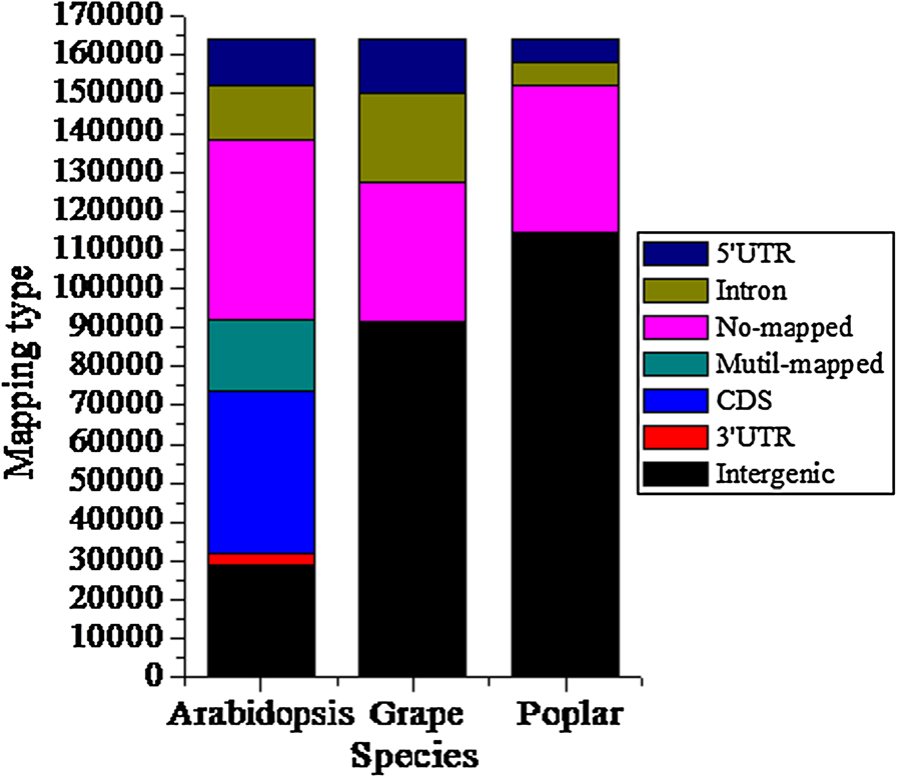
**Distribution of SSR reads mapping onto *Arabidopsis*, poplar, and grape genomes.** The x-axis includes three reference plants namely Arabidopsis, Grape species and Poplar; The y-axis indicates the numbers of sequences with SSRs mapped with the reference plants at various positions within genes/genomes. UTR = Untranslated region; CDS = Coding DNA sequence.

The distribution of SSRs from tree peony was analyzed based on *Arabidopsis*, grape, and poplar coding regions (Figure 6). Many sequences (25.6%) were mapped onto *Arabidopsis* CDSs, whereas only 0.1% and 0.3% were mapped onto CDSs of grape and poplar, respectively. A large number of sequences, 28.1%, 21.7%, and 22.9%, respectively, could not be mapped onto any *Arabidopsis*, poplar, or grape genomic region. More tree peony SSRs mapped to 5′ UTRs than to 3′ UTRs in the above three species, while 14,290, 23,133 and 5,982 SSR-containing sequences were mapped to introns of *Arabidopsis*, grape, and poplar genomes, respectively (Figure 6).

**Figure 7:**
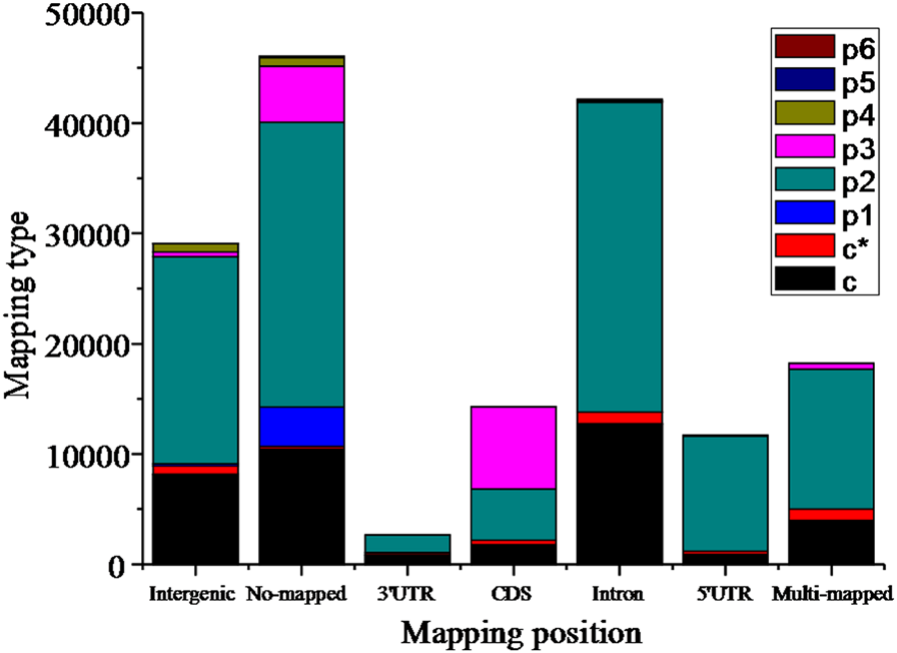
**Distribution of SSR reads with various repeat motifs mapping onto the *Arabidopsis *genome.** The x-axis indicates various position within genes/genome of Arabidopsis; The y-axis indicates the relative numbers with different repeat motifs mapped with various position of genes/genome of Arabidopsis. Abbreviations are: p1 = mono-nucleotide repeats; p2 = di-nucleotide repeats; p3 = tri-nucleotide repeats; p4 = tetra-nucleotide repeats; p5 = penta-nucleotide repeats; p6 = hexa-nucleotide repeats; c* = compound SSR without an interruption between two motifs; c = compound SSR with an interruption between two motifs; UTR = **U**n**t**ranslated **r**egion; CDS = **C**oding **D**NA **s**equence.

Unlike *Arabidopsis*, grape and poplar genomes have not been fully annotated; *Arabidopsis* was consequently used as a reference plant for further study (Figure 7). Most SSRs with mono-nucleotide repeats (94%) could not be mapped, and only 0.3% were observed in CDS regions. Among SSRs with di-nucleotide repeats, 44% mapped within 3′ UTRs, 5′ UTRs, introns, and CDSs, with 27% of these found in introns. Approximately 55% of SSRs with tri-nucleotide repeats mapped within CDSs, while most SSRs with tetra-, penta-, or hexa-nucleotide repeats mapped onto intergenic positions, or could not be mapped onto the *Arabidopsis* genome. With respect to c-type SSRs (i.e., without an interruption between two motifs), 33% mapped to introns, and 9% in total were mapped onto 3′ UTR, 5′ UTR, or CDS regions. In regard to c*-type SSRs (i.e., with an interruption between two motifs), 27% were mapped onto introns and 26% to intergenic regions (Figure 7). Among tree peony SSRs that were mapped to *Arabidopsis* CDS regions, those with tri-nucleotide repeats were the most abundant, followed by di-nucleotide repeat SSRs; in contrast, intergenic regions and introns contained more di-nucleotide and compound SSRs than did CDS regions (Table 4).

**Table 4:** Microsatellite distribution in different genomic regions of tree peony using the Arabidopsis genome as a reference

**Microsatellite distribution**				**Repeat motif**				
Mapping type	c	c*	p1	p2	p3	p4	p5	p6
Intergenic	8173	727	203	18797	410	754	2	10
No-mapped	10464	218	3560	25867	5039	766	24	80
3′ UTR	915	180	0	1534	14	0	0	0
CDS	1755	397	15	4674	7440	0	0	9
Intron	12760	1051	1	28034	130	135	0	0
5′ UTR	921	284	0	10409	59	0	0	1
Multi-mapped	3963	1024	0	12700	544	0	0	0

Compound SSRs are designated as follows: c* = no interruption between two motifs; c = interruption between two motifs; p1–p6 refers to the repeat motif (e.g., mono-nucleotide, di- nucleotide, etc.).

### Functional annotation of SSR-containing coding sequences

**Figure 8:**
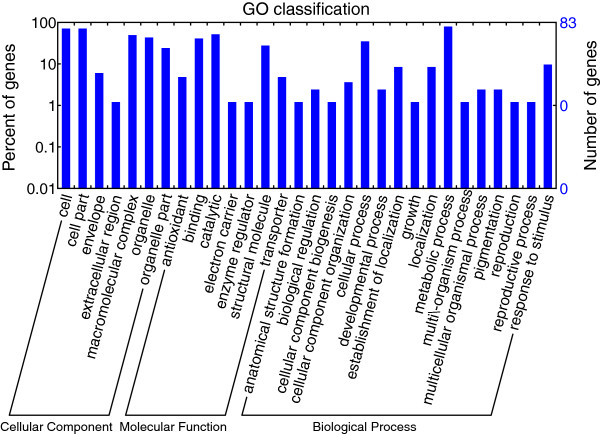
**GO classification of SSRs in coding regions, including the number/percentage of genes putatively involved in different subcellular functions.** The x-axis refers to different functional classes within a cell performing various functions. The y-axis indicates the percentage (left) or number (right) of genes within SSRs belonging to various functional classes.

Gene Ontology (GO) analysis was performed on sequences with SSRs mapping onto CDSs. Numbers of genes and GO classifications are displayed in Figure 8. Genes were classified into three major categories based on their sub-cellular function: cellular component, molecular function, and biological processes. Genes with functions related to cell and cell part (GO ID: 0044464), macromolecular complex (GO ID: 0032991), and organelle (GO ID: 0043231) were the most abundant genes in the cellular component category. The molecular function category was rich in genes associated with binding (GO ID: 0005488), catalytic activity (GO ID: 0003824), and structural molecules (GO ID: 0005198). Genes related to cellular process (GO ID: 0019941), metabolic process (GO ID: 0008152), and response to stimulus (GO ID: 0009607) were the most heavily represented in the biological process category.

### Validation of SSR assays

**Figure 9:**
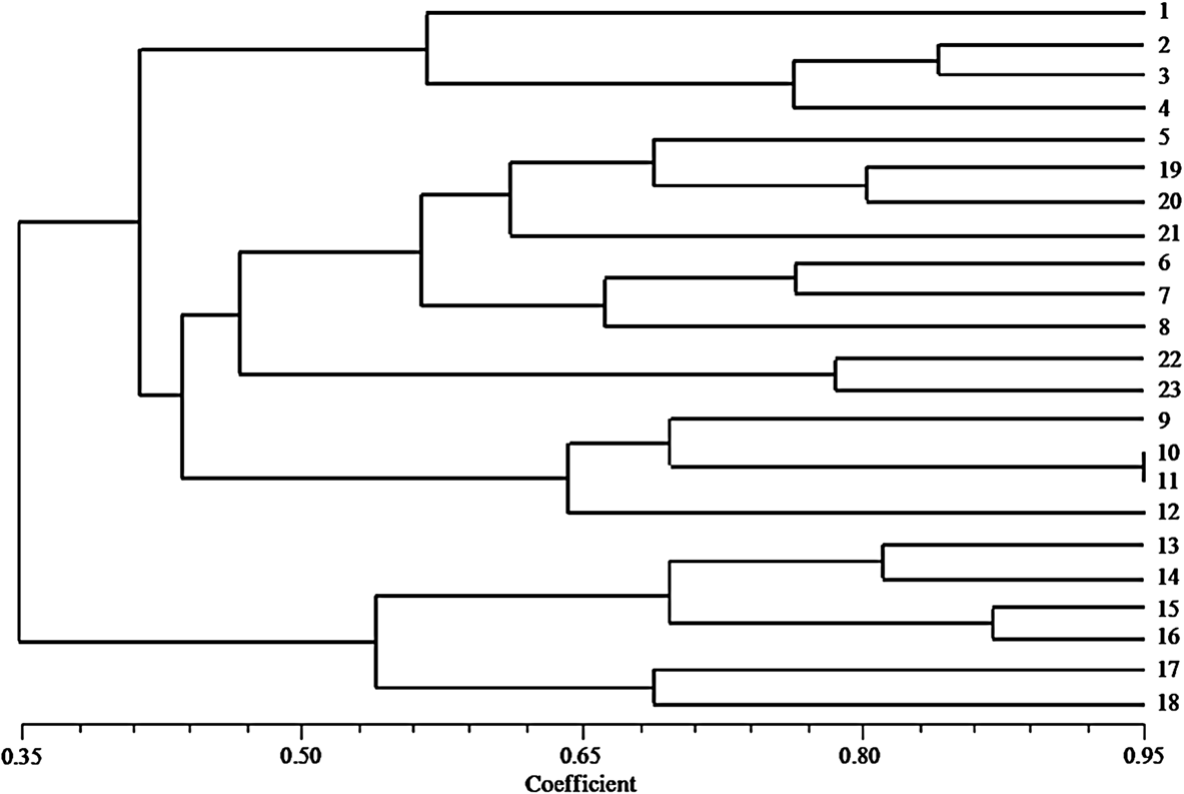
**UPGMA dendrogram constructed from 23 tree peony accessions using the markers developed in this study.** Numbers at leaf tips refer to accession code numbers listed in Additional file 2: Table S2.

A major advantage of the approach used in this study is the ability to obtain SSRs rapidly, thus greatly reducing the time and expense required to check for polymorphisms. In this study, 100 primer pairs were selected for validation using three tree peony accessions (Additional file 1: Table S1), and 24 primer pairs with high amplification effect were subsequently used for polymorphism analysis among 23 accessions (Table 5). The number of alleles per locus ranged from two to five; expected heterozygosity varied between 0.0850 and 0.7275, whereas observed heterozygosity ranged from 0.0000 to 0.8410 (Table 5). It has been confirmed that *P. rockii* and *P. ostii* are the ancestors of all 21 cultivars analyzed in this study. UPGMA analysis of SSR data resulted in cultivars ‘Yao Huang’ (1), ‘Dou Lv’ (2), ‘Shui Jing Bai’ (3), and ‘Liu Li Guan Zhu’ (4) from the Zhongyuan cultivar group clustering together, demonstrating their close genetic relationships to one another. Cultivars from the Japanese cultivar group—‘Taiyoh’ (10), ‘Shima Nisshiki’ (11), and ‘Gun Pou Den’ (12), derived from the Zhongyuan group, clustered with this latter group. All of these cultivars formed a major branch in the UPGMA dendrogram (Figure 9). Cultivars ‘Huai Nian’ (14), ‘Ju Yuan Shao Nv’ (15), and ‘Xin Xing’ (16) from the Xibei cultivar group clustered together, reflecting their close inter-relationships, and formed another branch in the UPGMA dendrogram (Figure 9). The results of cluster analysis of these SSR genotypes, consistent with known genetic relationships, are similar to results obtained using EST-SSR, TRAP, and SRAP markers [17,18], and confirm the usefulness of the SSR markers developed in this study.

**Table 5:** SSR loci amplified from 23 accessions of tree peony

**Locus**			**23 accessions**					
	**Repeat**	**Forward primer (5′-3′)**	**Reverse primer (5′-3′)**	**Size (bp)**	**Ta (ºC)**	***Na***	***He***	***Ho***
2A	TGG6	AACTGCGCTAGTCGTCCCCATAAAC	AAAGCCGCCTACAGAGGATGTTCAT	268	57	3	0.4647	0.0435
19A	CA16	TAACATCTCACTACCACTCAGGCGA	CATAAGGGTGATGATCATGTGGTTG	164	54.5	3	0.6493	0.0000
25A	TGT10	CAATCCCTTTTGTAATGCCCCTTTC	CAGGCTGTACTAGCAAAGGCTTCCA	215	54.5	3	0.5565	0.0000
26A	TTG7	TGGGCCCTACAAGTGATGATATTCC	ATGGAATCCAGGTTTGTGAATGTGA	245	54.5	3	0.559	0.0000
30A	CA13	TGTCATACCGACTTCGGCTAGGCTA	AAGGGTGATCGTGTGGTTGATGTTT	265	54.5	4	0.7275	1.0000
31A	CT11	AGCGCGTTTAATTGCTCTTACCTTG	CTCCCTCCTCTAACTCCATGCTTGC	303	54.5	3	0.6261	0.0000
36A	(TGG)6gctttggccggttcg(CTT)5	GACTGTAGTGATGGTGGTGGATTGG	AGCTTATGAACCCTGATGATGACGC	261	57	3	0.5797	0.0000
48A	CAG5	ACAGCGTCAGCAGACAGGAAGTACC	AAGAGTACCTGTCACCCCATCCAAA	364	57	4	0.5913	0.0000
49A	TGC5	TCTGGGTGATAGGTGGAGCTGGTGC	GGAAGACGCCCACAATGAAATCACA	314	57	4	0.6696	0.0000
50A	CA13	CACGGCTTTAAAATGCGTCTCAACT	AGGCTGGTGATAGTGTTGTTGATGC	252	54.5	4	0.5295	0.0000
53A	TCC5	CTCTTGTCAACCCCCACTGCCTCCT	GAAGGGACTTTCGCTGGAATCTGGC	353	59	4	0.6802	0.0000
54A	(CT)9(CA)14	TGTCGGGCGGTAAGTTTAGGGAAGA	CCACTTGGGTTCTGTTGGAGACTCG	388	59	3	0.5034	0.0435
56A	AC15	CAGGTGGCATTTTTGGCTTCTCTCT	TTGGCCCAATCACATGTAATCCCTC	388	57	3	0.5217	0.0000
58A	GCA6	TAGGATGACAAAGTGCAGGAAACCC	TGCTCAAACTCATCCTCAAGCTGTG	318	57	2	0.085	0.0000
59A	AC18	TACAACACTTCTCGCCTAACGCACC	AGACATGGTGCAAGTATGGGAGACG	270	59	3	0.4908	0.0000
63A	(TC)9(AC)17	CACCGCATATCTCCAACCTCACCTC	TTGGGTAGAGATAGGAGGTTGGGGC	277	59	3	0.6609	0.0000
65A	TGG5	CATACCTCCATCATGATGCTGCTGT	ATGAAGGCTCAGTAAGAACCTCGGA	355	57	3	0.3053	0.0000
73A	CAG5	CCATCTCAGGGTCAGGGTTCTCGTA	TAGAGTGTACCTTCACCCCCATCGG	375	59	4	0.6928	0.1379
78A	AC16	TATCAAATGGGGATGGTCTCCTCTT	AATTCTGCCACTATGAGCTCGATCT	314	54.5	5	0.6899	0.1579
79A	GCA5	AGAGGAAGTTTGAGGCCATCAGTCG	CAACTGTAGCCTTCTGTTCCTGCCC	367	57	2	0.4638	0.0000
80A	GTG5	AAGGTTATGGTGGCAGTGAAGATGA	ACCGTCGTACTACCACTTACAGCCG	207	54.5	4	0.6773	0.3043
87A	TG15	TGTAATCGATCGAGTTTCTTGGGTC	CCTAACACTCCACCACTAAGTCGCT	188	56	3	0.6261	0.0000
91A	(GT)9ttgta(TG)16	TCAGCCCCTAGCATAGAAGAATCCA	TCTCACTACCACCTACGCGATGTTC	384	60	3	0.6032	0.0000

Size = size of cloned allele; Ta = annealing temperature, Na = number of alleles; *He* = expected heterozygosity; *Ho* = observed heterozygosity.

## Discussion

The number of SSRs obtained in this study from tree peony was higher than that generated from other plants, including *Arabidopsis*, *Medicago truncatula*, *Oryza sativa* (rice), and *Sorghum bicolor* (sorghum) [1,19]. The frequency of A/T repeats present in tree peony was between dicots and monocots [18]. The percentage of tetra-, penta-, and hexa-nucleotide repeats observed in tree peony (8%) was higher than in *Sorghum* (5.4%), *Populus* (1.66%), *Medicago* (0.94%), rice (2.54%), *Brachypodium* (2.45%), and *Arabidopsis* (0.53%) [20].

The frequency of di-nucleotide repeats in tree peony was not consistent with that observed in *Brachypodium* by Sonah et al. [19]. Similar to rice, AG/CT repeats were well represented. AG/CT and AT/AT repeats were abundant in tree peony, accounting for 41.9% and 41.0%, respectively, of identified SSRs, while AT/AT repeats were more frequent in *Populus* (60.5%) and *Medicago* (59.9%) [19]. CG/CG repeats were relatively uncommon in tree peony, however, similar to *Populus*, *Medicago*, and *Arabidopsis*, suggesting that CG-rich motifs are the least preferred in dicot genomes. In human, *Caenorhabditis*, and *Arabidopsis* genomes, the most common di-nucleotide repeats are (AC)_n_, (AG)_n_, and (AT)_n_, respectively, demonstrating that different species have different motif frequency distributions.

With respect to tri-nucleotide repeats, AGC/CGT, AGG/CCT, and CCG/CGG have been observed more frequently in monocots than in dicots. A/T-rich repeats were the dominant tri-nucleotide SSRs in tree peony, similar to the results of Sonah et al. [19]. In tree peony, the sparseness or absence of CCG and ACG repeats may be due to highly mutable CpG di-nucleotide repeats, as evidenced in rice by the tendency of tri-nucleotide repeats, with few exceptions, to consist of various combinations of C and G. Transcriptional repression by DNA methylation depends on CpG density; CCG repeats may also be selected against by the requirements of the splicing machinery, with maintenance or absence of CCG possibly an active process [20]. The total absence of a particular repeat motif may indicate that the sequence is not preferred by the mechanism generating repeats or that strong selective pressure exists against repeated occurrence of particular sequences [20].

The characteristically short lengths of SSRs may have functional implications with respect to their evolution or the genes involved in plant physiology and development. In a previous study [4], rice SSRs were divided into two groups based on the length of SSR tracts and their potential as informative genetic markers: Class I microsatellites contained perfect SSRs ≥ 20 bp long and Class II microsatellites contained perfect SSRs 12–20 bp long. Class II microsatellites tended to be less variable because of less possibility of slipped-strand mispairing over the shorter SSR template. In tree peony, 85% of SSRs were categorized as Class I microsatellites and 1% as Class II microsatellites. Longer perfect repeats (Class I) have been determined to be highly polymorphic [21]. In future studies of tree peony SSRs, attention should focus on Class I microsatellites, with an emphasis on evaluation of polymorphism and its implications.

Length variation of repeated units may be due to differences in generation and fixation mechanisms of simple repetitive DNA. The inherent ability of a sequence to form alternative DNA conformations may be important for SSR generation, but does not explain differences observed among taxa. Enzymes or other proteins responsible for various aspects of DNA processing, such as replication and repair, and for chromatin remodeling, may be involved in the taxon specificity of microsatellite characteristics. It should be emphasized that not only do genomes differ in degree of repetitiveness [22], but also in preferred microsatellite types. In plant genomes, the frequent occurrence of repeat motifs of a particular sequence and length is the result of selection pressure applied on the specific motif during evolution [20]. The molecular mechanism responsible for the origin of microsatellites is still a subject of controversy, with many theories—such as replication slippage and unequal crossing-over—proposed to explain their occurrence [19,23,24]. The essential basis for species-specific accumulation of particular motif repeats, repeat lengths, and G/C content, which may influence unique microsatellite distribution patterns and evolution, is also still unclear. Variations in repetition purity and motif length enable site-specific adjustment of mutation rate and mutation effect, evidence indicating that common SSR alleles may offer potential selective advantages [25]. The increasing number of species with sequenced genomes should provide a foundation for the study of microsatellite evolution and even lead to discovery of the genetic/genomic role of microsatellites.

SSR frequency in monocot CDS regions is twice that of dicots [18]. It has been suggested that SSRs in different gene positions may perform varied functions. In animals, including mammals and other vertebrates, introns contain more poly (A/T) than poly (C/G) repeats. In *Caenorhabditis elegans*, however, intergenic regions show an interesting preference for poly (C/G) over poly (A/T) repeats [21], indicating that preferences may vary among organisms. In tree peony, the abundance of tri-nucleotide repeats mapping onto CDS regions was consistent with results found for the six species studied by Sonah et al. [19]. Tang et al. [26] examined SSRs in the *Arabidopsis* genome, and found that SSRs generally were preferentially located in upstream gene regions, especially 5′ UTRs; as in tree peony, tri-nucleotide repeats were the most common repeats found in coding regions. The accumulation of tri-nucleotide repeats in coding regions is primarily due to the triplet-repeat nature of codons [19]. The various numbers of repeats in coding regions are a potential source of quantitative and qualitative phenotypic variation [26]. SSRs in 5′ UTRs and CDSs may modify the expression or function of genes with which they are associated [26].

In rice, 80% of GC-rich tri-nucleotide repeats occur in predicted exons, while AT-rich tri-nucleotide repeats are distributed evenly across all genomic components. Di-nucleotide and tetra-nucleotide repeats are predominantly situated in noncoding—mainly intergenic—regions. (GA)_n_ repeats usually occur in regions with a balanced (close to 50%) GC content, favoring robust PCR amplification, whereas (CA)_n_ and (AT)_n_ are rare in gene-rich regions [4]. Tri- and hexa-nucleotide repeats have been shown to be the most common repeats in eukaryotic coding regions [20,27]. In our study, SSR-containing genes encoding for binding, catalytic, and structural molecules were abundant in the GO molecular function category, similar to results found in *Brachypodium*[19]. While such SSR-containing genes may perform multiple functions in tree peony, the importance of SSRs within genes remains to be further explored.

The SSR markers identified in this study should be useful for population genetic studies, and are potentially amplifiable across the genus. Plant genomes are complex, and contain large amounts of repetitive DNA, including microsatellites, which has immediate practical implications for the success of SSR marker development. Observed differential patterns of SSR marker distribution may be helpful for studying microsatellite evolution in a monocot-dicot system. SSR markers developed in this study have potential application to genomic research, marker-assisted breeding, DNA fingerprinting of genetic resources, molecular mapping of tree peony and related species, and map-based cloning of candidate genes. Hypervariable microsatellites are a useful source of polymorphic DNA markers for linking genetic maps with genomic sequences, and ultimately with phenotypic variation. They provide an opportunity to use SSR markers to investigate the wide range of genetic diversity that exists in wild relatives outside of the tree peony gene pool. Because SSRs are associated with vital functions and characteristics, such as transcription factor binding, RNA shape, DNA structure and packaging, and DNA length and orientation [24], the SSRs obtained in this study may be important for investigating plant genomic structural evolution and for providing insights into phenotypic variation in species during their evolution.

## Conclusions

This study represents the first application of next-generation sequencing for high-throughput microsatellite development in tree peony. The large size of the tree peony genome, approximately 16 G (data from private correspondence), hampers its sequencing, and the species is not highly amenable to transformation because of its woody characteristics. Consequently, the 237,134 microsatellites obtained in this study should be useful for marker-assisted breeding and functional characterization of genes related to trait formation. In addition, because the phylogenetic position of *Paeoniaceae* is still unresolved, the uncovered microsatellites may serve as a data resource for evolutionary studies in the family.

## Methods

### Plant materials

Leaves of tree peony (*Paeonia suffruticosa* Andrews) were collected from the Peony Germplasm Garden, Institute of Botany, Chinese Academy of Sciences (Beijing, China). Three cultivars—‘Liu li guan zhu’ , ‘Fu gui hong’ , and ‘Wu cai die’—were used for primer validation. Twenty-three accessions of tree peony were used for marker validation (Additional file 2: Table S2).

### Genomic DNA isolation, library preparation, and R454 sequencing

Total genomic DNA was extracted using the CTAB method [17]. Genomic DNA (500 μl; 600 μg) was fragmented with nitrogen at 45 psi for 2 min; 500–750-bp fragments were used for further study. Both fragment ends were polished and ligated to adaptors using T4 ligase. After PCR amplification of fragments with adaptor primers, selective hybridization was performed using eight biotin-labeled probes—pGA, pAC, pAAT, pAAC, pAAG, pATGT, pGATA, and pAAAT—and streptavidin-coated beads (Dynabeads; Invitrogen, Grand Island, NY 14072, USA) [28-31]. Library quality inspection and sequencing of clones was carried out as described by Yang et al. [31].

DNA (5 μg per plate) was sequenced on a Roche 454 GS FLX sequencer using Titanium reagents. Processing and analysis of sequencing data was performed with GS-FLX Software v2.0.01 (454 Life Sciences/Roche, Werk Penzberg82372, Penzberg, Germany). Raw sequences in SFF files were base-called using the python script sff_extract.py developed by COMAV (http://bioinf.comav.upv.es), and then processed to remove low-quality and adaptor sequences using the programs tagdust [32], LUCY [33], and SeqClean [34] with default parameters.

### SSR locus search and mapping

The program MISA (**Mi**cro**sa**tellite identification; http://pgrc.ipk-gatersleben.de/misa/) was used to identify reads and contigs containing SSRs. Criteria used for selection were a minimum of five repeats for simple motifs and three repeats for complex or imperfect repeats, a motif length of 2–10 bp, and, for compound SSRs, a maximum interruption distance of 100 bp between different SSRs. To facilitate SSR detection, only 1- to 6-nucleotide motifs were considered, and the minimum repeat unit was defined as 10 for mono-, 6 for di-, and 5 for tri-, tetra-, penta-, and hexa-nucleotides. SSR position, number of different repeat types, and length (motif bp × number of motifs) were analyzed using the ‘bespoke’ function in MISA [35] and plotted using Open Office Calc.

To map coding regions, all reads containing SSRs were compared against *Arabidopsis* (ftp://ftp.arabidopsis.org/home/tair), grape (http://www.genoscope.cns.fr) and poplar (ftp://ftp.jgi-psf.org) public databases using the program BWA-SW [36,37]. Map position was categorized as follows: 3′/5′ UTR, CDS, intergenic, intron, non-mapped, or multi-mapped. The repeat unit type (1–6, compound, or compound with interruption) was then determined.

GO annotation was conducted by searching against the Nr database using Blast2GO (*E*-value = 10^-6^) [38]. WEGO [39] and custom scripting were used to assign each GO ID to the related ontology entry.

### Primer acquisition and validation

Primer pairs for flanking sequences of each unique SSR were designed automatically using Primer3 [40], with target microsatellites containing at least five repeats and yielding PCR products of 80–500 bp. One hundred primer pairs were synthesized and used for validation (Additional file 1: Table S1). Screened primer pairs giving good amplification were subsequently used to characterize genetic diversity among 23 accessions of tree peony (Additional file 2: Table S2). PCR protocols and components were as described in [17], with modifications to annealing temperatures.

Number of alleles and expected and observed heterozygosities were calculated using POPGEN1.32 [41]. A dendrogram was constructed based on Nei’s unbiased genetic distances [42] using the unweighted pair-group method with arithmetic averages (UPGMA) as implemented in NTSYSpc-2.02 [43].

## Abbreviations

SSR: Simple sequence repeat; QTL: Quantitative trait locus; EST: Expressed sequence tag; CTAB: Cetyltrimethylammonium bromide; MISA: Microsatellite identification.

## Competing interests

The authors declare that they have no competing interests.

## Authors’ contributions

ZMG performed bioinformatic analysis, primer design and drafted the manuscript. JW and ZAL created the SSR sequences rich DNA library, and participated in 454 sequencing. LSW assisted in designing experiment and preparing the manuscript. HXR tested SSR markers. QYS participated in conceiving the study and the manuscript drafting. All authors read and approved the final manuscript.

## Supplementary Material

Additional file 1: Table S1 The primer pairs were designed for marker development. Click here for file

Additional file 2: Table S2 Accessions used in this study for marker validation. Click here for file
